# Clinical management of a ruptured intracranial aneurysm

**DOI:** 10.3389/fstro.2024.1450650

**Published:** 2024-09-24

**Authors:** Leonidas Trakolis, Athanasios K. Petridis

**Affiliations:** ^1^Department of Neurosurgery, St. Luke Hospital, Thessaloniki, Greece; ^2^Medical School, Heinrich Heine University Duesseldorf, Duesseldorf, Germany

**Keywords:** subarachnoid hemorrhage, ruptured intracranial aneurysm, delayed cerebral infarction (DCI), interdisciplinary work, intracerebral bleeding

## Abstract

**Background:**

Intracranial hemorrhage due to a ruptured aneurysm is one of the most serious neurosurgical emergencies. The patient mostly presents with severe headaches and neurological deterioration. A rapid diagnosis and an interdisciplinary approach play a major role in the fate of these patients. The treatment can vary from endovascular to surgical and must be carefully and individually planned. Neurovascular expertise and an interdisciplinary approach are of vital importance and obligatory for the best possible outcome.

**Methods:**

In this narrative review, we scrutinize the current literature and discuss the actual data and guidelines in order to emphasize the importance of the interdisciplinary expertise and approach in patients with ruptured intracranial aneurysm.

**Results:**

The current approach to patients with ruptured aneurysm is inhomogeneous and often ineffective due to internal disputes between different disciplines. Although there is plenty of literature and hard evidence to “show the way,” many still choose to base their decisions on personal experience or opinion.

**Conclusions:**

Every ruptured brain aneurysm should be approached in an interdisciplinary manor and treated according to the current evidence and guidelines.

## 1 Introduction

The prevalence of intracranial aneurysms is rather high, affecting about 5% of the population (Spetzler et al., 2013) while the annual incidence is 2%−3% (Wiebers, 2003; Petridis et al., 2017). They rarely occur in patients under the age of 20 and are more frequent in women and the elderly (Alessandro et al., 2013). The annual rate of aneurysmal subarachnoid hemorrhage (SAH) is 9 per 100.000 cases (Ma et al., 2023). Numerous factors have been associated with a high risk of rupture, including anatomical (Fung et al., 2019) and demographic features (Backes et al., 2016). Moreover, factors that trigger a sudden rise in blood pressure can lead to rupture (Vlak et al., 2011, 2012). Not all cerebral aneurysms rupture, and some remain undetected for years. However, 1.4% of cases do rupture every year, leading to demise or severe neurological deficits in most cases (Ma et al., 2023). Depending on the study, approximately 65% of these patients do not survive the rupture, while half of the survivors suffer lifelong disabilities (Nieuwkamp et al., 2009). As a result, patients with diagnosed but unruptured and untreated aneurysm often suffer from distress, depression, and anxiety (Li et al., 2017; Buijs et al., 2012; Dandurand et al., 2021). Nevertheless, in patients with a ruptured aneurysm, the symptoms are more severe (and sometimes permanent) and include cognitive and memory deterioration, speech disorders and sensory–motor impairment, in addition to the psychological symptoms mentioned above. This further reduces the quality of life (Alessandro et al., 2013; Zabyhian et al., 2018; Haug et al., 2009), regardless of how the aneurysm was treated (Schwyzer et al., 2015). Consequently, rapid diagnosis and proper treatment of a ruptured aneurysm are of great importance for the patient (Alessandro et al., 2013).

The course of intracranial aneurysm diagnosis and treatment has changed greatly over the years. The rapid development of medical technology and the availability of modern materials and devices have widened the options available to the treating clinicians (Tjoumakaris et al., 2024). Rapid admission to a specialized center (shorter transportation times than before) has reduced mortality (Tjoumakaris et al., 2024; van Lieshout et al., 2017). In addition, diagnostic procedures have evolved and are faster to perform and easier to interpret so that the median interval from hemorrhage to diagnosis can be <3 h (Germans R. M. et al., 2014). A three-dimensional rotational angiography helps the surgeon prepare them self better (Petridis et al., 2013), while the evolution of interventional neuroradiology has brought new, less invasive methods for the treatment of aneurysms that are surgically more challenging or less accessible (Lee et al., 2021).

With so many options, the choice of the proper method for each patient can be confusing. Ideally, an experienced neurovascular surgeon, together with the interventional neurologist/neuroradiologist, will study the available imaging and treat the patient individually, either with surgery or an endovascular procedure. Proper communication between the surgical and interventional specialists is decisive for making the right decisions concerning the diagnostic methods and the treatment options without losing any valuable time, especially in cases with multiple aneurysms (Mueller et al., 2011). However, good collaboration among the different specialists may be troublesome, and many cases will be treated based on one clinician's opinion/experience. This can lead to fatal mistakes and delays, which will affect not only the patient's (future) quality of life but, most importantly, their chance of survival. The type and timing of the therapy should be individualized to the patient according to the current evidence. Furthermore, not all methods are represented in every hospital, making the decision a Hobson's choice in some situations. In general, the ruptured aneurysm should be treated before vasospasm occurs, which means within the first 72 h. In cases with refractory intracranial hypertension of >20 cm H_2_O (angry brain), surgical clipping must be postponed since it is almost impossible to approach the aneurysm(s). In the case of intracerebral bleeding, an immediate hematoma evacuation should be performed, without spending time on EVD implantation or other secondary procedures. The initial diagnosis and treatment of the ruptured aneurysm is followed by a long period of time that is rather perilous for the patient. These patients belong in the intensive care unit during this time and should be treated with extra care from experienced clinicians, as the possible complications are many (Flemming et al., 1999). Cardiac symptoms are not rare, as the elevated catecholamine secretion in patients with subarachnoid bleeding can lead to myocardial necrosis and myocardial dysfunction (Petridis et al., 2017; Samuels, 2007). Therefore, SAH can be confused with cardiac infarction, and this misdiagnosis has proven to be highly fatal (van Lieshout et al., 2017). It is of utter importance for the cardiologist to be aware of ECG changes in SAH, which easily mimic a myocardial infarction. Another common complication is the derangement of the electrolytes in serum, either in the form of salt-waste syndrome or due to the syndrome of inappropriate antidiuretic hormone secretion (SIADH or Schwartz–Bartter syndrome) (Flemming et al., 1999; Ridwan et al., 2019; Loggini et al., 2021; Kieninger et al., 2021; Chen and Mitchell, 2016). Last but not least, vasospasm and delayed cerebral infarction are important and sometimes life-threatening complications that may (negatively) influence the prognosis of the patient.

As a result, patients with a ruptured intracranial aneurysm constitute an absolute emergency and their treatment carries many risks, even when the right decisions are made. A proper analysis of each patient in a matter of hours is surely a challenge but absolutely necessary and will determine the condition in which these patients will exit the hospital. In this narrative review, we present the flowchart of a patient with SAH due to a ruptured brain aneurysm from his presentation to the ER throughout the recovery process. We discuss the possible therapies and risks of each method as well as the possible complications during and after the procedure, attempting to point out the importance of the interdisciplinary approach according to the established standards.

## 2 Methods

To achieve that, we scrutinized the current literature about ruptured cerebral aneurysm of the last 25 years in order to collect the long-term results of the current treatments and the therapeutic protocols proposed from experts in the field. Included were peer-reviewed articles published in PubMed, Scopus and Google Scholar published (open-access or to us accessible) from 1999 until May 2024. Excluded were clinical studies with a small patient cohort (under 20 patients), inconclusive results or poor quality. The following key words and various combinations (according to the subject) of them were used in our search: “subarachnoid hemorrhage,” “ruptured brain/cerebral aneurysm,” “surgical clipping,” “coiling,” “endovascular treatment,” “vasospasm,” “delayed cerebral infarction,” and “ruptured brain/cerebral aneurysm management.” We provide an algorithm which will serve as an orientation for colleagues carrying out SAH treatment, although we strongly encourage that each case is dealt with individually according to the characteristics of the patient, the anatomy of the aneurysm and the experience of the treating physicians.

## 3 Symptoms and classification

Most of the patients with ruptured intracranial aneurysm present as emergencies within 2 h from the onset of symptoms with severe (thunderclap) headaches of sudden onset (about one-third of the patients) (Petridis et al., 2017; Alessandro et al., 2013; van Gijn et al., 2007; Claassen, 2022), nuchal rigidity (meningismus) because of meningeal inflammation (Kirkpatrick, 2002), nausea (with or without vomiting), and dizziness. Additionally, some patients have photophobia and altered mental status (confusion, agitation, reduction in consciousness) (Alessandro et al., 2013; Claassen, 2022). Increased intracranial pressure may lead to anisocoria (or mydriasis in more severe cases), seizures (Dennis et al., 2002; Little et al., 2007; Claassen et al., 2003), abnormalities in the ECG mimicking a myocardial infarct (Petridis et al., 2017; Alessandro et al., 2013) papilledema and downward deviation of the eyes (sunset eye sign), and intraocular hemorrhages (Terson's syndrome) due to obstruction of the central retinal vein by a distended meningeal optic nerve (Kirkpatrick, 2002). The intraocular bleeding can be vitreous, sub-hyaloid, both or retinal. Vitreous hemorrhages lead to vitrectomy since the visual acuity is significantly decreased. The rest of the intraocular hemorrhages seem to have a good resorption rate with satisfying visual recovery (Stiebel-Kalish and Turtel, 2004). Occasionally, the patient has ignored the symptoms for some time (especially if they only suffered from mild headache and nausea) and presents with ischemic neurological deficits because of the already present vasospasm (Claassen, 2022; Nussbaum et al., 1997). Further, a possible clot formation may lead to obstructive hydrocephalus (occlusus), while an impaired resorption of cerebrospinal fluid can lead to non-obstructive hydrocephalus (communicans). In both cases, the patient requires a lumbar or ventricular shunt, at least temporarily; otherwise, the consequences of the high intracranial pressure can be fatal. More specific symptoms can occur according to the location of the ruptured aneurysm. For instance, dysphasia or motor or sensory disorders may point to eloquent regions of the brain, oculomotor palsy to posterior communicans aneurysm, and nystagmus and ataxia to cerebellum and brainstem damage (Edlow and Caplan, 2000). About 30% of all patients with aSAH develop some form of hydrocephalus, while 10% will have some intraocular hemorrhage (Petridis et al., 2017).

The most widely used classifications for ruptured aneurysms are those of Hunt and Hess (1968), Fisher et al. (1980), and the World Federation of Neurological Surgeons (WFNS) (Rosen, 2005; CG, 1988). They classify patients according to their neurological condition and symptoms (Hunt and Hess and WFNS classification) or according to the amount of blood in the brain (Fisher's classification). Nevertheless, over time, more sophisticated classifications have been suggested based on imaging studies tailored to the individual patient (Alwalid et al., 2021; Pritz, 2011; Cebral et al., 2011), or even based on applying a machine learning algorithm to patient data, including hemodynamic and morphologic characteristics of cerebral aneurysms (Tanioka et al., 2020). In this way, it has been possible to predict the risk of further bleeding or re-rupture and the general prognosis of the patient.

## 4 Diagnostic procedures

Most SAH patients present with severe headache, nausea and vomiting, followed by altered mental status and loss of consciousness. Furthermore, increased intracranial pressure can cause seizures, focal neurological symptoms, and cranial nerve deficits (Alessandro et al., 2013). Such cases constitute a real emergency that must be treated immediately. The first diagnostic step is a neurological examination, followed by a proper imaging. After admission to the ER (emergency room), a specialized team of anesthesiologists, neurologists and neurosurgeons will try to stabilize the vital parameters of the patient and take a quick medical history of the symptoms and time of onset. The primary imaging is usually a native (non-contrast) CT scan, performed by a (neuro-)radiologist, and may show a subarachnoid hemorrhage, sometimes combined with an intracerebral bleeding, providing a sensitivity and specificity that approaches 100% during the first 6 h after headache onset and 90%−100% during the first 24 h (Petridis et al., 2017; Dubosh et al., 2016). The sensitivity of the CT scan declines after this time due to progressive blood dilution by the flow of cerebrospinal fluid (Dubosh et al., 2016). In most cases, subarachnoid and/or intracerebral bleeding. Usually, the location and distribution of the bleeding are enough for the experienced clinician to suspect the cause; however, it is not enough for a definite diagnosis (Byyny et al., 2008).

In agreement with the neurosurgeon, the radiologist will usually perform a CT angiography with 3D projection in order to identify any vascular disorders of the brain vessels. The aneurysm(s) will be identified and related (or not) to the bleeding and symptoms of the patient. A CT angiography may reveal the cause of the bleeding. In patients who must be operated on immediately (due to the amount of bleeding, signs of hydrocephalus or increased intracranial pressure), the experienced surgeon can proceed without it in order to avoid severe brain compression and damage due to loss of time (Batjer and Samson, 1991). When the patient shows signs of ventricular ectasia, the placement of an external ventricular drain is indicated irrespective of the method of aneurysm occlusion [GCS of 12 or less (Petridis et al., 2017)]. If the patient's life is not threatened by brain swelling and high intracranial pressure, a digital subtraction angiography (DSA) of the intracranial arteries will be performed to enlighten the clinicians about the anatomy of the ruptured aneurysm and to show further smaller aneurysms and/or perforating vessels (Chappell et al., 2003). In some cases, however, depending on the experience of the surgeon, the DSA is not always necessary if there are convincing findings in the CT angiography, especially when embolization is out of the discussion (Kouskouras et al., 2004; Sebök et al., 2021; Anderson et al., 1999).

If the imaging shows no signs of bleeding, a lumbar puncture is usually suggested to exclude signs of blood and, more importantly, the presence of siderophages in the spinal canal due to sedimentation (Petridis et al., 2017; Farzad et al., 2013). The sensitivity of siderophages increases after 12 h; however, in the ultra-early period after SAH, their presence may be scarce. Xanthochromia in CSF can be a useful indicator (Goyale et al., 2016) in CT-negative SAH diagnosis. Spectrometry may help detect bilirubin and oxyhemoglobin in CSF, while CSF cytology can detect siderophages even weeks after the event (Martin et al., 2015). Nevertheless, blood can enter the CSF sample during the lumbar puncture itself (Martin et al., 2015; Walton et al., 2022), but still the false positive rate for SAH is under 5% (Walton et al., 2022). A lumbar puncture is also necessary to rule out major differential diagnoses such as a meningitis (Brunell et al., 2013).

## 5 Discussion

Intracerebral hemorrhage from a ruptured aneurysm is a multifaceted condition. From the first moment, and for the rest of the patient's life, it is important to be treated by clinicians from different disciplines (neurosurgeon, neurologist and neuroradiologist); during hospitalization, the intensive (critical) care specialists are a valuable part of the team as well. From the moment the patient is admitted to the ER, the interdisciplinary expertise will improve the patient's prognosis and reduce the number of complications (Carvi et al., 2009). The teamwork in such cases is so important that, according to Voellger et al. (2019) the results of clipping and coiling are similar, regardless of the method chosen, as long as there is teamwork. The results are similar in patients with unruptured aneurysms, where fewer complications and better results can be achieved when there is effective collaboration between neurosurgeons and interventional (neuro-)radiologists (Gerlach et al., 2007).

### 5.1 Endovascular treatment

In many cases, especially in older patients (over 70), wide-neck aneurysms or when the ruptured aneurysm is not safely surgically accessible, endovascular treatment is the right choice. During this method, to limit the blood circulation, the ruptured aneurysm will be occluded with platinum spirals (coils) causing an unorganized thrombus and granulation tissue formation. It is favored in saccular aneurysms and aneurysms of the posterior circulation with a neck-to-dome ratio of more than 1:2. When the coiling alone is not sufficient, a stent (Phan et al., 2016) or balloon remodeling (Pierot et al., 2020; Wang et al., 2016) may be required in order to achieve complete occlusion or reduce the recanalization rate. The recurrence (reperfusion) rates are about 30% (Ravindran et al., 2020; Diana et al., 2022), varying according to the method used and the grade of occlusion achieved during the primary treatment (Pierot et al., 2022; Li et al., 2020). This bloodless method of treating cerebral aneurysms has gathered a lot of attention over the last 30 years, leading to rapid progression and development of further endovascular options. In cases of complex aneurysm configuration or dissecting aneurysms, the double microcatheter technique may be utilized, while in aneurysms with a wide neck, there is the option of a flow diverter (Ravindran et al., 2020; Briganti et al., 2015) and intrasaccular flow disruptors (Kaschner et al., 2020; Kortman et al., 2023; Van Rooij et al., 2017). These will divert the flow of the blood away of the aneurysm, leading to thrombosis (Kaschner et al., 2020, 2019). These methods were found to provide higher complete occlusion rates (80%−90%), with only a 10% re-treatment rate (Kortman et al., 2023; Van Rooij et al., 2017; Lubicz et al., 2014), although the follow-up period was relatively short. In any case, the endovascularly treated aneurysms require longer follow-up due to the higher re-perfusion rates in comparison to the clipped aneurysms (Higashiguchi et al., 2021). Nevertheless, in cases of stent- or device-assisted coiling or flow diversion, the use of dual antiplatelet therapy is required. This increases the risk of intracranial bleeding when hydrocephalus occurs and an EVD or permanent shunt-system placement is necessary (Hudson et al., 2019). Should the patient develop an infarction of the middle cerebral artery (MCA) and require a decompressive craniotomy, the surgery will be performed under dual antiplatelet treatment with a significantly higher bleeding risk. Furthermore, the double antiplatelet therapy may complicate the course of the patient, increasing the risk of intracranial hemorrhage (Ryu et al., 2015). On the contrary, some studies suggest that the dual antiplatelet therapy reduces the risk for vasospasm and the general thromboembolic risk (Higashiguchi et al., 2021; Sun et al., 2020; Nagahama et al., 2018).

Molyneux et al. (2002), with the International Subarachnoid Aneurysm Trial (ISAT), compared neurosurgical with endovascular treatment of ruptured intracranial aneurysms in over 2,000 patients. This trial showed a significant advantage for short-term (1-year) survival, free of disability, for the endovascular group, although it was based mostly on high-grade subarachnoid hemorrhage (SAH) patients with small anterior circulation aneurysms. On the contrary, 2 years earlier, Koivisto et al. (2000) reported comparable clinical and neuropsychological outcomes after early surgical and endovascular treatment of ruptured intracranial aneurysms. Some years later, a new trial (ISAT II) included patients otherwise not suitable for the ISAT (Darsaut et al., 2013). The efficacy of clipping in patients with ruptured MCA aneurysm was proven superior to the endovascular treatment (Darsaut et al., 2021b; Steklacova et al., 2016). The meta-analysis of Zhou et al. (2023) found a complication rate of MCA aneurysm occlusion with a flow diverter of 20%, which is far higher than that with surgical clipping. Thirteen years after the publication of the first ISAT results, Molyneux et al. (2015) presented an 18-year follow-up study. They concluded that despite the higher rebleeding rate for endovascular coiling, this risk is low, and the probability of disability-free survival (in patients without rebleeding) during the first 10 years is significantly greater in the endovascular group than in the neurosurgical group, coming to a more balanced conclusion in comparison with ISAT. Lanzino et al. (2013) published a meta-analysis of the three high-quality, prospective, controlled trials, concluding better outcomes for the endovascular treatment in patients amenable to either therapeutic strategy. A comparable meta-analysis from Shao et al. (2019) showed better occlusion rates for the surgically clipped patients, with lower rebleeding rates, but a significantly increased risk of poor outcome.

A couple of years ago, an Italian multicenter study (Scerrati et al., 2021) reported 250 aneurysms of the posterior circulation treated endovascularly with a complete occlusion rate of 65%. About 75% of the ruptured aneurysms of the posterior circulation that were coiled showed an immediate complete occlusion, while 27% showed a re-perfusion in the follow-up examination, leading to secondary procedures in 15% of cases. Almost half (41%) needed a secondary flow diverter, while 29% required a stent-assisted re-coiling. The rest could be treated with simple coiling, remodeling or microsurgical clipping. Furthermore, during the procedure, a posterior communicans artery (PComA) thrombosis occurred in 9% of the cases, which was pharmacologically successfully treated, leading to recanalization, while <1% of the aneurysms ruptured during the procedure without major neurological consequences for the patient. According to Froelich et al. (2020) in aneurysms of the anterior circulation, the occlusion rate was 82%, the recurrence rate was 11% and 13.5% had to be re-treated. Although the complete occlusion rate in aneurysms of the posterior circulation was lower (77%), the re-treatment rate remained the same as that with aneurysms of anterior circulation.

The most common complications during the endovascular occlusion of a ruptured aneurysm are thromboembolic/ischemic events (2%−8%). Therefore, periprocedural dual antiplatelet therapy must be initiated (Meyer et al., 2023) even though this leads to higher intracranial hemorrhage risk. In the cases of non-respondence or an inadequate effect, fibrinolytics, and mechanical thrombectomy come into consideration. Less common are the side-branch occlusions, with a rate of 1.4%. These are mostly due to overlapping flow diverters, occurring more frequently in the posterior circulation (Chalouhi et al., 2014). Once again, adequate antiplatelet preparation and sparing use of individual flow-diverting devices may prevent the problem. Iatrogenic vascular injury and/or rupture of the parent artery can cause an intraprocedural hemorrhage in 5.4% of cases and post-procedural hemorrhage in 3.6% of cases (Ryu et al., 2015). If a perforation is suspected during endovascular treatment, an extravasation of contrast at the site of perforation can be identified in the angiography. Indirect signs are the abrupt increase in systemic blood and/or intracranial pressure and Cushing reflex (concomitant bradycardia). In this case, the anticoagulation must be reversed (use of antidote if available) and the blood pressure must be held under control. Platelet transfusion may help as well. If this happens during a procedure on the cavernous ICA, it can lead to iatrogenic carotid cavernous fistula. Although this kind of complication is very rare (0.8%), the parent artery may have to be sacrificed if transvenous embolization will not help. Last, but not least, malposition or migration of the flow-diverting stent (Abdalkader et al., 2020) mostly leads to thromboembolic events (Al-Mufti et al., 2019). In 12% of cases, the device has to be removed to prevent complete occlusion of the parent artery, while sometimes the issue is the prolapse. However, these do not constitute serious issues for the patient in most cases (Pema et al., 2013). Despite the periprocedural risks, there can also be some delayed complications. About 7% of patients develop an intracranial hemorrhage during the first 48 h, while in few cases, this can develop weeks or months later (Hu et al., 2014). Further, in 3.2% of cases, a delayed rupture of the occluded aneurysm may be observed (Weill et al., 2013) and in one-third of cases, there can be a delayed occlusion of the parent artery, partially due to patient incompliance with antiplatelet medication (Lubicz et al., 2010). Regardless of the occlusion method, there is a 7.6% chance of *de novo* aneurysm recurrence in patients with treated aneurysmatic subarachnoid hemorrhage (Vourla et al., 2019).

### 5.2 Neurosurgical treatment

Multiple or giant aneurysms, aneurysms of the anterior circulation or those with a narrow neck are often treated surgically. Before opening the dura, an external ventricular drainage or a lumbar drainage is placed to achieve brain relaxation (Yang et al., 2023). Draining of the CSF starts after the dura is opened, and CSF is removed deliberately until the brain is relaxed enough to allow a safe approach to the aneurysm-bearing vessel. The neurosurgeon will reach the vessel with the aneurysm and surgically prepare the surroundings, acquiring a better view of the perforating vessels and more space for the clipping procedure, as well as proximal control in case of an intraoperative rupture. A temporary clipping of the proximal vessel may be necessary in order to dissect the aneurysm without the risk of re-rupture. In 20%−30% of cases, more than one aneurysm will be identified in the brain, making it hard to identify which one is the bleeding source (Hadjiathanasiou et al., 2020; Rosi et al., 2021). There are studies which have identified anatomical markers that can specify which aneurysm is the ruptured one in such cases (Fung et al., 2019). The complete occlusion of the aneurysm without narrowing the parenting vessel is usually confirmed with a micro-Doppler device and intraoperative near-infrared indocyanine green video (ICG) angiography (Raabe et al., 2003). Each method has its own advantages and disadvantages, as analyzed by Petridis et al. (2014).

Clipping is widely indicated and associated with high occlusion rates (95%), low re-treatment rates (1%−5%), and low morbidity (3%−5%) (Lee et al., 2021). For challenging cases where clipping (clip reconstruction in giant aneurysms) of the aneurysm is not possible, Spetzler et al. (1984), among many other experts, suggested the extracranial–intracranial bypass. The parent artery can then safely be ligated while the brain perfusion remains intact (Shi et al., 2011). The morbidity, however, is higher than that with simple clipping (5%−10%). More recently, intracranial–intracranial bypass was suggested for better obliteration of the aneurysm, although the procedure is more complex and requires more surgical expertise (Sanai et al., 2009). If neither of these options are possible, wrapping with muslin can provide some protection from further rupture of the aneurysm (Baldoncini et al., 2020; Schartz et al., 2021) prior to endovascular treatment (Lee et al., 2021; Choudhri et al., 2013). Although the rushed development of the medical technology has offered many kinds of clips for surgeons to use, there are still cases where none will fit or the neck of the aneurysm will be lacerated during the clipping. In this case, a trapping procedure could be an acceptable compromise (Fukuda et al., 2014). This should be carried out with a bypass or an anastomosis of the artery ends.

Recently, the Barrow Ruptured Aneurysm Trial brought some interesting results (Spetzler et al., 2019). Although the clinical outcomes for patients with ruptured aneurysm of the posterior circulation were superior for the endovascular group at 1 year, this difference diminished after that time. Furthermore, the complete occlusion rates and the rates of re-treatment were better in the surgically treated group, while there were no significant differences in the poor outcome and death rates between the two groups. Clipping of ruptured intracranial aneurysms has been shown to be associated with increased morbidity in various studies. However, a systematic review of over 12,000 procedures concluded that only 6 of the 36 most common adverse events during surgery are associated with poor outcome (Muirhead et al., 2021). This result is in agreement with McLaughlin and Bojanowski (2004), who reported a procedure-related complication rate of 20% after surgical clipping, including hemorrhagic contusions in about 6% of the cases that eventually disappear, and a small aneurysm remnant in 5%. They concluded that surgical complications could be overlooked because of the good functional status of the patients after surgery. Similarly, the 15% rate of ischemic complications during endovascular treatment of MCA aneurysms is high, but the patients remain mostly asymptomatic and thus it does not affect the outcome (Zhou et al., 2023). As a matter of fact, these intra-procedural complications are manageable, leading to low morbidity.

The crossover rate from endovascular treatment to surgical clipping in these studies is also a point that cannot be neglected. All the studies can be interpreted based on the bias of the particular expert, but the advantages and disadvantages of each method are clear, since the limitations of the embolization become more obvious over time. ISAT was overoptimistic about the potential of end-vascular treatment, and ISAT 2 and BRAT showed more balanced conclusions, as expressed by the meta-analyses. Long-term results for the endovascular treatment option have yet to be published, whereas those for the surgical treatment have been known for decades.

### 5.3 Therapeutical indications

**Figure 1:**
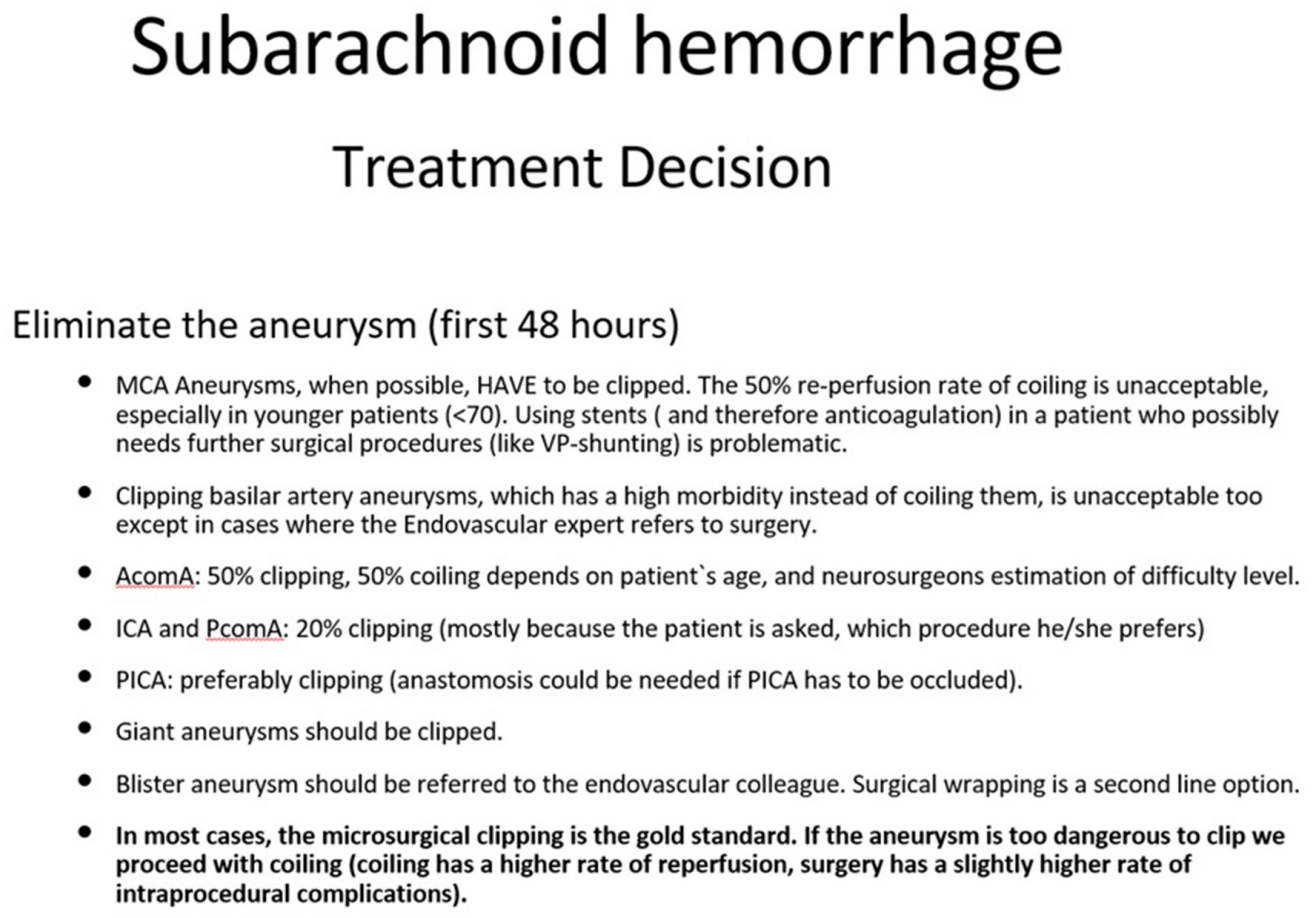
How to treat the aneurysm. This figure represents the author's opinion on how to treat an aneurysm. Many factors play a role in the treatment strategy: occlusion rates, rebleeding risk, aneurysm anatomy, age, post procedural management, etc.

Although endovascular treatment is not always the best choice for some aneurysms, the in-hospital mortality at institutions where this is available was significantly lower (Johnston, 2000) emphasizing the importance of interdisciplinary treatment. Consequently, surgical clipping may have higher intraprocedural risks, but, concerning the occlusion rates, it remains the gold standard especially for younger patients. A narrow neck can be an indication for coiling and clipping as well. A giant aneurysm can frequently cause compression of adjacent nerves and structures, causing perifocal edema, and should be clipped whenever possible (Bohl et al., 1977; Hassan, 2013; Durner et al., 2018). Basilar artery aneurysms should be coiled almost exclusively, and MCA aneurysms should be clipped. In some cases, where the surgical risk is too high, the availability of alternative treatment methods may prove pivotal. Older patients, for instance, are often multimorbid, so the method with the lower morbidity (endovascular treatment) will be favored (Schwartz et al., 2018). Therefore, not all aneurysms of the medial cerebral artery are appropriate for clipping (Suzuki et al., 2009) and not all aneurysms of the posterior cerebral artery have to be coiled (Schwartz et al., 2018; Steiger et al., 1999). In more complicated cases, such as the infectious aneurysms, the participation of an infectious disease specialist in the primary treatment is strongly recommended (Hamisch et al., 2016). In case of blister aneurysm, the case is handed over to the interventional neuroradiologist to be treated with a flow-diverter stent (Kaschner et al., 2020; Rasskazoff et al., 2010; Kan et al., 2020; Madjidyar et al., 2023). In case this is not possible, the neurosurgeon will proceed with Teflon wrapping of the aneurysm (Kubo et al., 2006; Baldoncini et al., 2020). We summarized the suggested procedure for the most common cases in Figure 1.

### 5.4 Evidence based treatment of complications of SAH

Not all patients with a ruptured cerebral aneurysm will be in bad clinical condition. Nevertheless, each of them should be treated in the intensive care unit before and after aneurysm occlusion. A tight surveillance of the blood gases, the electrolyte balance, urea and glucose are important, while the cardiovascular condition should be continuously monitored as well. If circumstances allow, permanent monitoring of the intracranial and arterial pressure can help evaluate the autoregulation mechanisms of the patient. According to the severity of the bleeding (Schmieder et al., 2006) and delayed infarction (Jaeger et al., 2007), it is not uncommon to observe a disturbance of the cerebral autoregulation. When the neurological condition is adequate (the patient is awake and cooperative), then more invasive monitoring is obsolete. Alternatively, in patients with a progressive deterioration of the GCS (Glasgow Coma Scale), intracranial pressure monitoring with an external ventricular drain is strongly suggested. However, intracranial pressure (ICP) alone is not a reliable indicator of prognosis as it changes with age (Czosnyka et al., 2005; Pedersen et al., 2018) and is easily influenced by systemic factors.

More sophisticated monitoring methods can also be used, if available. The most common are the monitoring of brain tissue oxygen and the lactate/pyruvate ratio (LPR) measurement through a microdialysis catheter in the brain or cerebrospinal fluid (CSF). Both measurements are indicators for the extent of brain injury due to anaerobic metabolism and predictors of possibly poor outcome (Jaeger et al., 2007; Sahuquillo et al., 2014; Lazaridis, 2014; Zahra et al., 2019). However, lactate and the lactate-to-pyruvate ratio should not be used as independent indicators of brain metabolism since lactate can be elevated in patients with SAH because of hyperglycolysis rather than hypoxia (Sahuquillo et al., 2014; Oddo et al., 2012). Hyperglycolysis reflects the attempt of the brain to restore energy supply by utilizing more glucose to improve the long-term outcome (Zahra et al., 2019; Oddo et al., 2012). Brain damage is not only the result of anaerobic metabolism. During the first 21 days after aneurysm rupture, the arteries of the brain can develop vasospasm, a serious condition that will be discussed separately. Transcranial Doppler examinations are part of the routine examination for the evaluation of brain perfusion, whereas regular CT or MR perfusion scans can detect smaller brain ischemia more efficiently. Needless to say, sophisticated cerebral monitoring only makes sense after occluding the aneurysm, in order to monitor for vasospasms and ischemia. Otherwise, time will be unnecessarily lost placing such devices, causing stress to the patient, and the information gained before aneurysm occlusion does not have any value in this emergency situation.

Seizure prophylaxis is recommended in patients with ruptured MCA aneurysm (Raper et al., 2013), high-grade SAH, intracranial hypertension, hydrocephalus, and cortical infarction, as well as patients presenting with seizures (high-risk patients) (Tjoumakaris et al., 2024). If possible, a cEEG should be performed first. Antiepileptic treatment is mostly used when seizures have manifested or are suspected, as it may reduce the patient's consciousness level (Dewan and Mocco, 2015). However, in patients with good awareness, a prophylactic anticonvulsive therapy may improve the final outcome (Chou et al., 2015). After the treatment of the ruptured aneurysm, low-dose heparin is used for prophylaxis against thrombosis, providing additional benefits (Khattar and James, 2018; Xi et al., 1998). Thus, early treatment of the problem can spare the patient from thromboembolic complications.

Spreading depolarizations (SDs) are waves of neuronal depolarization and are associated with worse tissue injury and poor clinical outcome in patients with SAH (Hartings et al., 2009; Sugimoto and Chung, 2020; Lauritzen et al., 2011). They are associated with vasospasm and DCI, and, recently, several animal models have partially enlightened their pathogenesis. Together with excitotoxicity, seizures and epileptiform discharges have been reported to occur very frequently in association with DCI development after SAH (Suzuki et al., 2022). Horst et al. (2023) identified spreading depolarization and angiographic spasm as separate mediators of DCI, while they confirmed that the amount of subarachnoid blood on the initial CT scan predicts DCI (Horst et al., 2023; Macdonald and Schweizer, 2017). In most (neuro-)intensive care units, scalp EEG is a standard procedure that gives valuable information about seizures. However, in order to detect spreading depolarization, invasive neuromonitoring is needed. The gold standard is electrocorticography (ECoG) with a subdural strip electrode (Sivakumar et al., 2022). DISCHARGE-1, a phase 3 trial, recently investigated the impact of spreading depolarizations in patients with SAH and proposed their use as a detector of reversible neurological deficits and impending infarcts in unconscious patients (Dreier et al., 2022). In this way, it is possible to identify the patients who are most likely to benefit from targeted management strategies and novel therapeutic approaches (e.g., N-methyl-d-aspartate receptor antagonists and phosphodiesterase inhibitors) (Berhouma et al., 2022).

One of the worse complications to occur in patients where therapy is delayed, is the re-rupture of the aneurysm, provoking active/extensive intracranial bleeding (Klisch et al., 2003). It occurs in about 12%−16% of these patients (Germans M. R. et al., 2014), mostly during the 1^st^ h after the first bleeding (Tack et al., 2019). Almost all rebleeds occur during the first 24 h (Germans M. R. et al., 2014) and have a high mortality rate (Roos et al., 2000; Vergouwen et al., 2016). Early occlusion is definitely beneficial for the patient and improves the prognosis (Klisch et al., 2003; Germans M. R. et al., 2014); however, ultra-early treatment (within 24 h) has shown less benefit than expected (Linzey et al., 2018). In the case of EVD placement before aneurysm occlusion, the draining volume per hour must be controlled, since a high flow rate of >58 ml within 6 h can induce re-rupture in 50% of cases (Van Lieshout et al., 2017).

Last year, the AHA/ASA (American Heart Association/American Stroke Association) published the new guidelines for the management of patients with aneurysmal subarachnoid hemorrhage (Hoh et al., 2023) refreshing the guidelines published in 2022. They emphasized the importance of rapid admission of the patients in specialized centers, as a rebleeding of an untreated aneurysm is associated with high mortality rates. These results are in accordance with a German study published in 2017, which analyzed the delay in patients with SAH arriving at the appropriate facility (van Lieshout et al., 2017). It also identified factors influencing the delay in moving a patient from a primary care hospital to the neurovascular center, with misdiagnosis of cardiac infarction being a significant factor, leading to an increase in mortality. In 2019, Doukas et al. (2019) analyzed the misdiagnoses of acute subarachnoid hemorrhages. These studies were predecessors of the AHA/Stroke guidelines, which proved their conclusions independently in their Top 10 take-home messages (point 1). Early initiation of enteral Nimodipine is beneficial, while prophylactic antiepileptic therapy during the 1^st^ days after SAH is suggested only in high-risk patients (i.e., intubated patients) or in those with new-onset seizures. Furthermore, the authors highlighted the importance of an interdisciplinary approach before, during and after occlusion of the bleeding aneurysm—as well as on discharge when identifying the extended needs of the patient (physical, cognitive, and behavioral)—to improve their quality of life as much as possible.

### 5.5 Management of vasospasm

The most common vascular complication is cerebral vasospasm, first described in the 1980s (Kassell et al., 1985; Kassell, 1982). It affects 30%−40% of patients with aSAH, leading another 20%−30% to a delayed cerebral infarction (DCI) (Li et al., 2019). It usually appears between the 4^th^ and 14^th^ days after the bleeding and can cause severe cerebral infarction (Rumalla et al., 2021). The exact mechanism of intracranial vasospasm is unknown; however, the dominating theory suggests that the vessels react to the metabolites of the blood cell disintegration in the subarachnoid space. This leads to (partial/complete) closure of the vessels, causing delayed cerebral infarction (DCI), with a catastrophic impact on the patient's outcome (Claassen, 2022). In these cases, induced hypertension may be a reasonable and effective option to obliterate the symptoms of DCI (Haegens et al., 2018; Jafari et al., 2023) although recent research doubts its efficacy (Loan et al., 2018). For prevention of delayed cerebral ischemia through vasospasms, oral nimodipine application is the most effective method used routinely around the globe (Schwarting et al., 2023; Allen et al., 1983; Stevens et al., 2009). When managing the patient in the intensive care unit, practices that are widely accepted include reducing the blood pressure and lowering the body temperature with cooling measures if necessary (Stevens et al., 2009). Hemodynamic monitoring is mostly performed through a central venous catheter, but central arterial catheters are also used routinely in some centers, especially in more challenging cases.

Vasospasms can be macro-vasospasms of the main vessels in the circle of Willis, or micro-vasospasms in small cortical vessels. Macro-vasospasms can be detected by transcranial Doppler sonography, whereas micro-vasospasms require more sophisticated diagnostic methods like CT perfusion and endovascular angiography (Romenskaya et al., 2022), often remaining undetected until infarcts or symptoms occur. Conventional angiography is the gold standard for the evaluation of vasospasms, providing the possibility for endovascular rescue procedures [intra-arterial infusion of calcium channel blockers and balloon angioplasty (Labeyrie et al., 2019; Findlay et al., 2015; Darsaut et al., 2021a)] when necessary. In most institutions, transcranial Doppler examination is standard during the high-risk phase (Vergouwen et al., 2016), as it is an inexpensive, portable and repeatable method. If, for instance, a progressive acceleration of ≥120 cm s−1 to <200 cm s−1 in the mean flow velocity of the middle cerebral artery is reported, further diagnostics (CT angiography or DSA) should be taken into consideration. However, the CT perfusion scan plays a crucial role in predicting delayed cerebral infarction and should be performed generously (Cremers et al., 2014; Dietrich et al., 2020; Mir et al., 2014).

Endothelin-1, a potential vasoconstrictor, is released by the vascular endothelium due to the SAH. For this reason, Idorsia produced a fast-acting endothelin A (ETA) receptor antagonist (clazosentan) for the prevention of vasospasm. The first trial (CONSCIOUS-1) showed a statistically significant decrease in moderate and severe vasospasm depending on the applied dose, without serious side effects (MacDonald et al., 2008). Similar results were reported in two phase 3 studies in Japan (Endo et al., 2022). Unfortunately, CONSCIOUS-2 failed to confirm these promising results, leading the CONSCIOUS-3 study to an abrupt completion (Meglio, 2023; Macdonald et al., 2010, 2013). Additionally, another phase 3 study (REACT) (Bruder et al., 2022) also revealed disappointing results, vanishing any hope for clazosentan, as announced by Idorsia itself (Meglio, 2023).^1^ Further methods to reduce or prevent vasospasm have also been suggested. In a meta-analysis, lumbar CSF drainage was proposed as an efficient way to prevent vasospasm and consequently delay cerebral infarction (Lee et al., 2022). The anti-inflammatory and neuroprotective role of heparin in SAH and brain oedema is also remarkable (Lauritzen et al., 2011; Suzuki et al., 2022).

An interdisciplinary approach is also in this case of vital importance. The neurosurgeon, the interventional (neuro-) radiologist and the anesthesiologist in the intensive care unit must be in continuous communication to notice the problem, react rapidly and proceed to further treatment (spasmolysis, intensification of the systemic treatment, etc.) to reduce the risk of cerebral infarction and brain oedema. The presence of both disciplines for the accurate treatment of aneurysm is not only necessary but obligatory.

### 5.6 The importance of interdisciplinary collaboration

Unfortunately, not every hospital can offer 24/7 interdisciplinary expertise for patients with subarachnoid hemorrhage, especially in developing countries. In Africa, for instance, the resources, expertise and medical technology available are very limited, making proper training of the clinicians even more essential (Djientcheu et al., 2024). In addition, the lack of diagnostic tools and endovascular expertise further compromises the treatment of ruptured aneurysms (Dokponou et al., 2021). Our experience in Africa showed that the quality of aneurysm treatment highly depends on the expertise of the surgeons and the anesthesiologists rather than on the equipment and instruments available. By establishing several training centers in every country, the time from aSAH diagnosis to treatment can be significantly shortened, hence decreasing the mortality of those patients (van Lieshout et al., 2017; Doukas et al., 2019). Furthermore, special attention should be given in countries with remarkably low aSAH rates due to the lack of diagnosis and treatment options. This could significantly improve the morbidity and mortality rates in these countries, which are still notably higher compared to developed countries (Tetinou et al., 2021).

Conflicts in healthcare are not news, and various studies have tried to understand and solve them (Carvi et al., 2009; Voellger et al., 2019). Poor communication and insufficient teamwork may lead to medical errors. Overworked clinicians may avoid the slightly more time-consuming case discussion with other disciplines, while sometimes the mutual respect and collegiality are missing. With the intension to solve this problem, the interprofessional education collaborative of Washington, DC, highlighted specific values to consider when working with other disciplines: the “patient first” regime, teamwork and the desire for quality care (Carvi et al., 2009). Good communication, as well as altruism and mutual respect, should be added. These qualities must be incorporated in the education of medical students, already a common practice in some countries (Gerlach et al., 2007; Scerrati et al., 2021).

**Figure 2:**
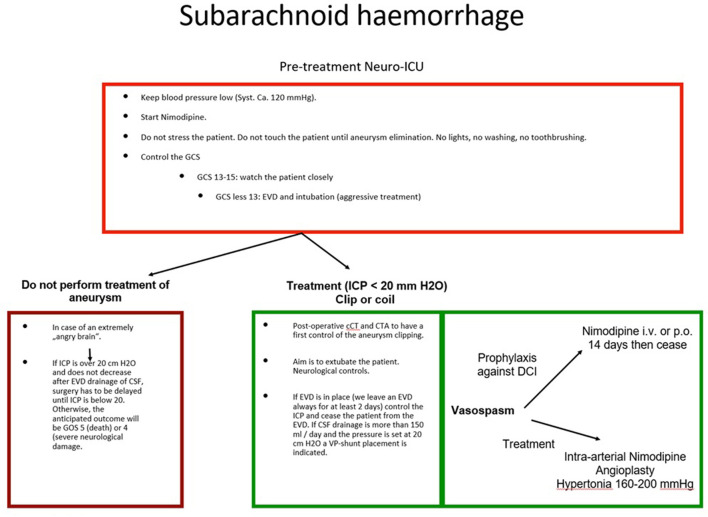
Management of SAH. The flow chart summarizes the management of patients with SAH after aneurysm rupture. The pre-treatment of the aneurysm and the post-treatment strategies are illustrated.

Nowadays, a greater number of clinicians are involved in patient treatment, and the responsibilities must be optimally distributed, especially if more than one discipline is involved. The availability of interdisciplinary expertise is, however, not a guarantee that a proper case discussion will take place before treatment. Not every hospital offers multidisciplinary neurovascular therapy, but even if they did, it would not mean that they are equally specialized. It is not uncommon that the dominant department, or the one where the patient was initially admitted, will make every decision without consulting the rest of the specialists involved, especially decisions concerning the primary treatment of the ruptured aneurysm. This dominance may superficially reduce the friction between disciplines, but it does not guarantee correct therapy according to the protocol. Even worse, clinicians frequently ignore the established methods or indications and base their decisions on personal experience or opinion. This can and will deteriorate the atmosphere of cooperation between colleagues and will lead to more solitary decisions that do not take into consideration the knowledge and experience of the other disciplines. The wrong decision in the modality of treatment can lead to an incomplete occlusion with complications that will probably influence the course of the patient. Naturally, the clinicians ignored in first place may refuse to help overcome some problems until they are shown equal respect in the decision-making process. A further delay in decision-making predisposes a worse prognosis for the patient, of course, and the failed or inadequate initial treatment will complicate any further procedures, increasing the risks involved. One discipline alone is not able to “pull out all the stops” in most cases, and this can reduce the spectrum of examinations or treatments carried out. Even the correct interpretation of the examinations performed may be challenging, leading to further mistakes. Needless to say, a therapy plan that follows established protocols and is in general interdisciplinary agreement sets a solid argument against any legal doubt. All this considered, it is exigent to have interdisciplinary contribution in treating patients with ruptured cerebral aneurysm. Indeed, experience is crucial in many instances but is not enough to guarantee that everything is done right in situations where any mistake can irreparably harm the patient. In the flow chart (Figure 2), we provide a guide for the management of SAH.

## 6 Conclusion

A ruptured cerebral aneurysm is a serious condition that must be treated immediately. The treating center must possess the necessary materials and expertise in order to make the right decisions and offer high-quality, interdisciplinary treatment. When it comes to a human life, the personal opinions and experiences of individual clinicians are not as important as interdisciplinary expertise. Neurosurgeons, neurologists, (neuro-)radiologists and anaesthesiologists must work together and set aside any personal conflicts. This will reduce the necessary time for important decisions to be made and provide proper treatment in each case. We strongly encourage specialized neurovascular centers where all the necessary experts are available 24/7 and collaborate selflessly based on current evidence.

## Footnotes

^1^*Idorsia announces the results of REACT a Phase 3 study of clazosentan in patients following aneurysmal subarachnoid hemorrhage*. Available at: https://www.globenewswire.com/news-release/2023/02/06/2601762/0/en/Idorsia-announces-th%5B%E2%80%A6%5Dpatients-following-aneurysmal-subarachnoid-hemorrhage.html (accessed May 13, 2024).
